# Serological Testing for COVID-19, Immunological Surveillance, and Exploration of Protective Antibodies

**DOI:** 10.3389/fimmu.2021.635701

**Published:** 2021-08-19

**Authors:** Luis A. Peroni, Jessica M. Toscaro, Camila Canateli, Celisa C. C. Tonoli, Renata R. de Olivera, Celso E. Benedetti, Lais D. Coimbra, Alexandre Borin Pereira, Rafael E. Marques, José L. Proença-Modena, Gabriel C. Lima, Renata Viana, Jessica B. Borges, Hui Tzu Lin-Wang, Cely S. Abboud, Carlos Gun, Kleber G. Franchini, Marcio C. Bajgelman

**Affiliations:** ^1^Brazilian Biosciences National Laboratory, Center for Research in Energy and Materials, Campinas, Brazil; ^2^Medical School, University of Campinas, Campinas, Brazil; ^3^Laboratory of Emerging Viruses (LEVE), Department of Genetics, Evolution, Microbiology and Immunology, Institute of Biology, University of Campinas, Campinas, Brazil; ^4^Experimental Medicine Research Cluster (EMRC), University of Campinas, Campinas, Brazil; ^5^Molecular Sciences Undergrad Program, University of São Paulo, São Paulo, Brazil; ^6^Research Division, Dante Pazzanese Cardiology Institute, São Paulo, Brazil; ^7^Infectious Diseases Section and Hospital Infection Control Committee, Dante Pazzanese Cardiology Institute, São Paulo, Brazil; ^8^Faculty of Pharmaceutical Sciences, University of Campinas, Campinas, Brazil

**Keywords:** COVID-19, SARS-CoV-2, immunoassay, nucleocapsid, 3CL, seroneutralization

## Abstract

Serological testing is a powerful tool in epidemiological studies for understanding viral circulation and assessing the effectiveness of virus control measures, as is the case of SARS-CoV-2, the pathogenic agent of COVID-19. Immunoassays can quantitatively reveal the concentration of antiviral antibodies. The assessment of antiviral antibody titers may provide information on virus exposure, and changes in IgG levels are also indicative of a reduction in viral circulation. In this work, we describe a serological study for the evaluation of antiviral IgG and IgM antibodies and their correlation with antiviral activity. The serological assay for IgG detection used two SARS-CoV-2 proteins as antigens, the nucleocapsid N protein and the 3CL protease. Cross-reactivity tests in animals have shown high selectivity for detection of antiviral antibodies, using both the N and 3CL antigens. Using samples of human serum from individuals previously diagnosed by PCR for COVID-19, we observed high sensitivity of the ELISA assay. Serological results with human samples also suggest that the combination of higher titers of antiviral IgG antibodies to different antigen targets may be associated with greater neutralization activity, which can be enhanced in the presence of antiviral IgM antibodies

## Introduction

SARS-CoV-2 is a single-stranded RNA virus that belongs to the *Betacoronavirus* genus. The virus genome encodes 27 proteins. The virus envelope of SARS-CoV-2 consists of a phospholipid bilayer containing structural proteins such as spike protein (S), membrane protein (M), and envelope protein (E). The capsid also harbors the most abundant structural nucleocapsid protein (N) that plays a critical role in genome packaging. The S protein is a surface glycoprotein that decorates the virus envelope and mediates binding to cell surface receptors and particle internalization on target cells. The membrane protein acts as a scaffold that interacts with the envelope protein that mediates membrane bending and cleavage to generate virus particles (1–3). Non-structural proteins participate in biological processes such as viral replication and pathogenesis (4). These proteins are translated as polyproteins that are cleaved by the 3CL protease to generate functional proteins (5).

The S and N proteins are the most used antigens for detecting antiviral antibodies (6–9). Assays targeting the detection of N antigen may show greater sensitivity than assays targeting the S antigen. Previous data revealed a longer persistence of antibodies generated against N protein in human serum compared to other SARS-CoV structural proteins (7, 10, 11). The IgM antiviral antibodies appear in the acute phase of viral infection and can be detected about 3 to 6 days of symptom onset (9), while IgG antibodies can be detected after 2 to 3 weeks of symptom onset (6, 9). The gold standard assay for COVID-19 diagnosis is the PCR assay, which detects the genome of the SARS-CoV-2 virus (12); however, the detection rate may be less than 70%, in the case handling problems and sample collection or even the loss of the window of detection of viral replication (9, 13). The use of serological assays in conjunction with the PCR test may improve COVID-19 diagnosis (9, 12). In contrast to the PCR test, which has a narrow range of time to detect the virus during the infectious condition, serological tests make it possible to detect antibodies even after the loss of symptoms and resolution of the infection (14). The combination of viral antigens may enhance antiviral serological detection assays (15).

Human antibodies may have the ability to neutralize the SARS-CoV-2 virus (16, 17); however, data in the literature report the coexistence of antiviral IgG antibodies in individuals with active infection by SARS-CoV-2 for more than 45 days (18). This observation raises questions about the correlation between the presence of antiviral antibodies and protective immunity against infections or reinfection by SARS-CoV-2. Therefore, it is relevant to investigate new strategies based on serological tests that may suggest protective immunity by neutralizing antibodies. In this work, we explore results of serological testing with SARS-CoV-2 viral antigens N, 3CL, and associations with virus neutralization potential.

## Materials and Methods

### Viral Propagation and Inactivation

SARS-CoV-2/SP02/human/2020/BRA isolated in Brazil (GenBank accession number MT126808.1), kindly provided by Prof. Edison Luiz Durigon (USP-SP, Brazil), were propagated in Vero CCL81 cells (BCRJ, #0245). Experiments with SARS-CoV-2 infectious particles were performed in a Biosafety level-3 (BSL-3) facility from the University of Campinas (UNICAMP). Further experiments with inactivated particles were performed in a BSL-2 facility, at CNPEM, after SARS-CoV-2 inactivation by 30 min of UV exposure. The validation of the inactivation procedure was performed by inoculation of treated supernatants into Vero cell culture. The cell culture media was harvested and tested by plaque-titration assay and qPCR to check the absence of virus.

DENV and ZIKV (MR766) were propagated in Vero CCL81 cells in a BSL-2 facility, at CNPEM, and titered by plaque assay. The virus produced in the supernatant of the cell culture was used in immunization assays, without inactivation. Adenovirus preparations were grown in HEK293 at a BSL-2 laboratory at CNPEM. The produced Adenovirus was a non-replicant viral vector, lacking E1 and E3 genes (19). The Rhinovirus preparation was kindly provided by Dr. Clarice Arns from UNICAMP.

### Mice Immunization

The 6- to 8-week-old female BALB/c mice were acquired from CEMIB (Centro Multidisciplinar para Investigação Biológica, Campinas, SP) and maintained, housed in groups of five in propylene cages, under specific pathogen-free conditions, fed a standard laboratory diet, and given water *ad libitum*. All experimental procedures were performed in accordance with the ethical regulation established by the Brazilian College of Animal Experimentation and approved by the Animal Experimentation Ethics Committee of the CNPEM.

Four each antigen (SARS-CoV-2, DENV, ZIKV, Adenovirus, and Rhinovirus), five female BALB/c mice were intraperitoneally injected with 100 μl as follows: first dose of 1 × 10^4^ PFUs in Freund Complete Adjuvant (FCA). Seven days later, a second dose was administered, replacing FCA by FIA (Freund Incomplete Adjuvant). Furthermore, 14 days after the first dose, the blood was collected through cardiac puncture or from the venous sinus (retro-orbital bleeding), and the serum was separated and stored at −20°C for further analysis.

### Human Samples

Serum samples and PCR samples were collected from the staff of Dante Pazzanese Hospital. The samples were collected by healthcare professionals with approval from the Ethics Committee of the Dante Pazzanese Hospital. All handling of human samples for ELISA assays was performed at a BSL-2 facility at CNPEM. The human serum was heat inactivated at 56°C for 30 min.

### Cloning Procedures and Plasmids

The SARS-CoV-2 RNA was isolated from virus particles with the QIAmp viral RNA mini kit (Qiagen, USA) and reversely transcribed to cDNA with the High Capacity Reverse Transcription Kit (Thermo, USA). The N sequence (GenBank: QIG56001.1) was amplified from cDNA samples using primers SC2-protN28182-F (5’-AGTCTTGTAGTGCGTTGTTCG-3’) and SC2-protN29566-R (5’-ATAGCCCATCTGCCTTGTGT-3’) and cloned into pGEM-T Easy (PROMEGA, USA), generating plasmid pGEM-SC2-N. The N sequence was reamplified from pGEM-SC2N with forward 5’-AACAAGCTAGCATGTCTGATAATGGACCCCAAAATCAG-3’ and reverse 5’-GGTCTGCGGCCGCTTAGGCCTGAGTTGAGTCAGCACTGCT-3’ primers and subcloned into the *Nhe*I/*Not*I sites of a pET28a-TEV vector carrying a 6xHis-tag and TEV protease cleavage site at the N-terminus.

The Mpro sequence (GenBank: QIG55993.1) was amplified from a synthetic gene with optimized codon usage for bacterial expression (GenScript, USA) using the forward 5´-CGCCCATGGCCCGCGGATCCTCGGCAGTGCTGCAATCAGGATTTAGGAAAATGGCTTTCCCCTCG-3´ and reverse 5´-CGTCAGTGCAGCGGGGTGACGTTCCAAGGACCCCATCATCATCATCATCATTAAAAGCTTCGG-3´ primers and cloned into the *Nco*I/*Hind*III sites of pET28a (Novagen, USA). The designed construct carries an Mpro cleavage site (SAVLQ/SGFRK) at the N-terminus and a modified PreScission cleavage site (SGVTFQ/GP) preceding a 6xHis-tag at the C-terminus (20).

### Protein Expression and Purification

The N protein was expressed in *Escherichia coli* BL21 (DE3) cells (Novagen, USA) and purified by metal-affinity and size-exclusion chromatography. Cells were grown at 37°C under agitation (200 rpm) in LB medium containing kanamycin (50 mg/L) to an optical density (OD_600nm_) of 0.8. Recombinant protein expression was induced by the addition of 0.1 mM isopropyl-thio-β-d-galactopyranoside (IPTG) for 16 h at 25°C. After centrifugation, cells were resuspended in lysis buffer (50 mM sodium phosphate, pH 7.6, 300 mM NaCl, 10% v/v glycerol, and 1 mM phenylmethylsulfonyl fluoride) and incubated on ice with lysozyme (0.1 mg/ml) for 30 min. Bacterial cells were disrupted by sonication and the soluble fraction was loaded on a 5-ml HiTrap Chelating HP column (Cytiva, USA) previously equilibrated with the same buffer. Proteins were eluted using a linear gradient (20 to 500 mM) of imidazole at a flow rate of 1 ml/min. Eluted fractions containing the N protein were pooled, concentrated, and loaded on a HiLoad 16/60 Superdex 200 column (Cytiva, USA), previously equilibrated with 10 mM Tris, pH 8.0, and 100 mM NaCl, at a flow rate of 0.5 ml/min.

The Mpro protease was produced as previously described (21). BL21(DE3) cells carrying the Mpro construct were cultured in YT medium supplemented with kanamycin (100 mg/L) and incubated at 37°C under agitation (200 rpm). When the OD_600nm_ reached 0.8, the temperature was lowered to 16°C and protein production was induced with 0.5 mM IPTG for 16 h. Bacterial cells harvested by centrifugation were resuspended in 20 mM Tris, pH 7.8, and 150 mM NaCl and lysed by sonication on ice. The cell lysate clarified by centrifugation was loaded on a 5-ml HisTrap column (Cytiva, USA) previously equilibrated with the same buffer. Bound proteins were eluted with a linear imidazole gradient (0 to 500 mM) in 20 mM Tris, pH 7.8, and 150 mM NaCl. Fractions containing the Mpro protein were pooled, mixed with a GST-tagged PreScission protease (5:1 molar ratio), and dialyzed overnight at 4°C in 20 mM Tris, pH 7.8, 150 mM NaCl, and 1 mM DTT. The dialyzed fraction was loaded onto a GSTTrap (Cytiva, USA) connected to a HisTrap column to simultaneously separate the 6xHis-tag and PreScission protease from the cleaved Mpro, which was further dialyzed overnight in 20 mM Tris, pH 8.0, and 1 mM DTT. The suspension was loaded on a HiTrap Q column (Cytiva, USA) equilibrated with the same buffer. Bound proteins were eluted with 20 mM Tris, pH 8.0, 1 M NaCl, and 1 mM DTT, with a linear gradient of NaCl (0 to 500 mM). Fractions containing the Mpro protein were concentrated and subjected to a size-exclusion chromatography on a HiLoad 16/600 Superdex 75 column (Cytiva, USA) equilibrated with 20 mM Tris, pH 7.8, 150 mM NaCl, 1 mM EDTA, and 1 mM DTT.

Protein purity was analyzed by SDS-PAGE (**Supplementary Figure S5**), and protein concentration was determined by absorbance at 280 nm using the molar extinction coefficient calculated from the amino acid composition. Protein samples were concentrated and stored at −80°C.

### Enzyme-Linked Immunosorbent Assay (ELISA)

ELISA was performed in 96-well plates coated with 1 μg/ml of virus antigen. When indicated, plates were also coated with 0.1 μg/ml of antigen or 1:250 dilution of inactivated virus. Plates were coated overnight at 4°C, washed, and incubated with the indicated serum dilution for 2 h at 37°C. The anti-mouse IgG-HRP secondary antibody (Sigma, USA) was added 1:10,000 and incubated at 37°C for 1 h. The anti-human IgG-HRP secondary antibody (Sigma, USA) was added 1:30,000 and incubated at 37°C for 1 h. The TMB substrate (Thermo Scientific, USA) was added, reactions were stopped with 1 N HCl, and plates were read at 450 nm.

### Plaque Reduction Neutralization Test

Plaque reduction neutralization test (PRNT) was performed in 24-well plates with a confluent monolayer of Vero CCL-81 (ATCC) maintained in DMEM supplemented with 10% fetal bovine serum (FBS) and 1% v/v penicillin/streptomycin. Serial dilutions (1/10, 1/20, 1/40, and 1/80) of SARS-CoV-2 antibody-positive and -negative human serum were heat inactivated for 30 min at 56°C before use. Serum samples were incubated with 100 PFU of SARS-CoV-2 for 1 h at 37°C. Antibody–virus complexes (250 µl) were added on Vero cell cultures and incubated at 37°C for 1 h. The virus–serum inoculum was removed after the adsorption, and 1 ml of 1% w/v carboxymethyl cellulose (CMC) overlay medium containing 5% FBS and 1% penicillin/streptomycin was added per well. Plates were incubated for 72 h at 37°C at 5% CO_2_. Plates were washed and fixed in 8% w/v PFA for 1 h at room temperature and subsequently stained in 1% w/v methylene blue. Virus plaque-forming units (PFU) were counted and compared to wells infected with SARS-CoV-2 in the absence of antibody to evaluate percentual of PFU reduction.

## Results

### Serum of Animals Immunized With Inactivated Viral Particles Are Reactive to Recombinant N and 3CL Antigens

**Figure 1:**
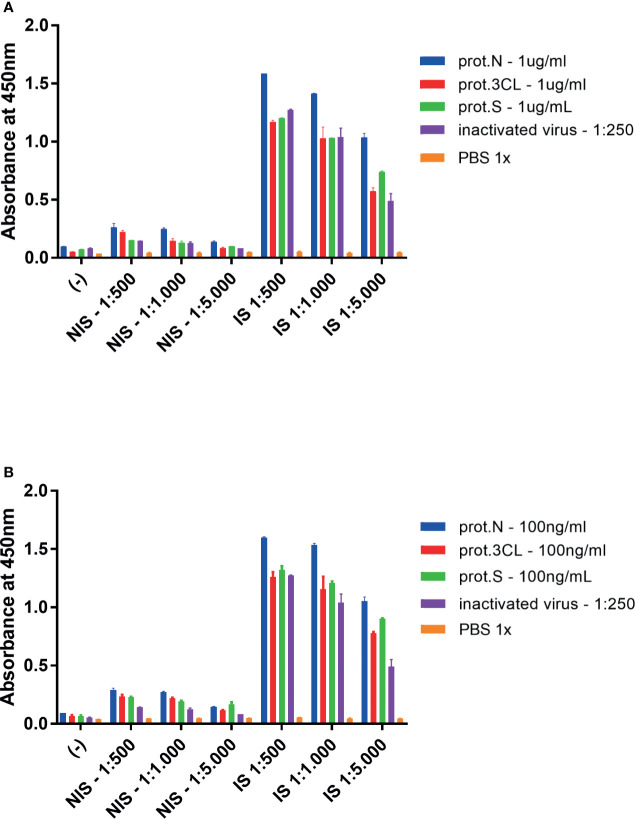
ELISA assay for detection of mice antiviral antibodies. Mice were immunized with inactivated SARS-CoV-2 virus, and serum samples were collected with 14 days. **(A)** Plates were coated with 1.0 mg/ml of indicated viral antigens or 1:250 of inactivated virus. **(B)** plates were adsorbed with 100 ng/ml of indicated viral antigens or 1:250 of inactivated virus. Target antigens: prot.N (N protein), prot.3CL (3CL protein), prot.S (S protein), inactivated virus 1:250 (inactivated virus diluted 1:250), PBS 1x (1X PBS diluent as negative control). Two-way ANOVA, *p* < 0.05 for IS against NIS for all target antigens, but PBS 1x, in all dilutions. Representative experiment of two independent experiments. The serum of five animals was pooled for each condition.

The genes encoding viral proteins N and 3CL were cloned from the cDNA of SARS-CoV-2 in bacterial expression vectors. The proteins were expressed, purified, and characterized (**Supplementary Figure 5**). ELISA (Enzyme-Linked Immunosorbent Assay) was performed, in which these proteins were used as antigens in two concentrations (1 μg/ml and 100 ng/ml) on test plates. The inactivated viral particles diluted 1:250 were used as a control. Next, the serum from animals immunized with SARS-CoV-2 virus particles (IS) and the serum from non-immunized animals (NIS) were added. The result showed that antiviral IgG antibodies could be detected in immunized animals in three dilutions of serum (1:500, 1:1,000, and 1:5,000), for both antigenic targets in experiments in which plates were coated with 1 μg/ml of antigen (**Figure 1A**) and 100 ng/ml (**Figure 1B**).

### Cross-Reactivity Assay Shows High Antigen Selectivity for Anti-SARS-CoV-2 Antibody Detection

**Figure 2:**
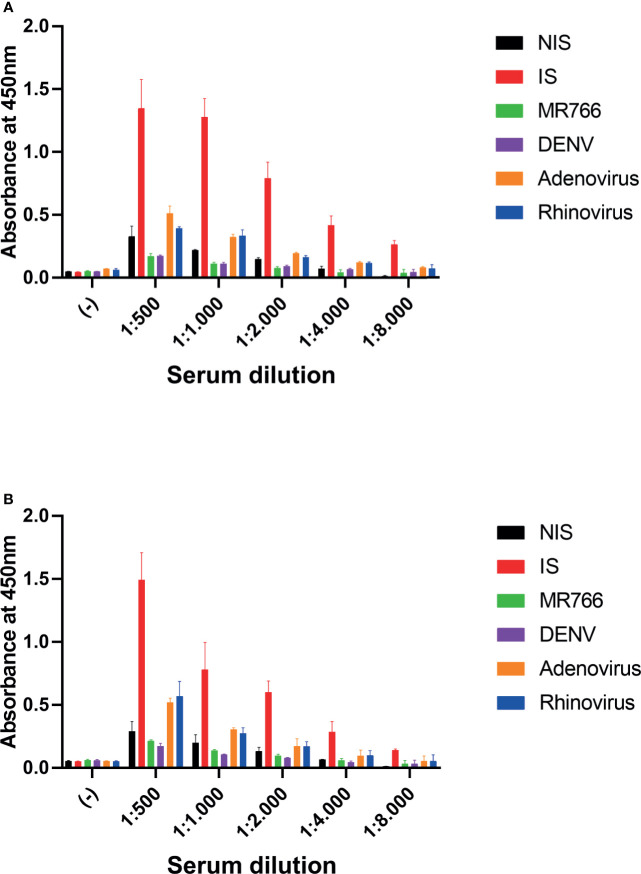
Cross reactivity assay. Serum samples at indicated dilutions were incubated with antigen-coated plates. **(A)** Plates were coated with N protein. **(B)** Plates were coated with 3CL protein. (−) negative control, no serum; 1:500, 1:1,000, 1:2,000, 1:4,000, and 1:8,000: serum dilution; NIS, non-immune serum; IS,immune serum of mice immunized with inactivated SARS-CoV-2; MR766, immune serum of ZIKV challenged mice; DENV, immune serum of DENV challenged mice; Adenovirus, immune serum of Adenovirus challenged mice and Rhinovirus: immune serum of Rhinovirus challenged mice. Two-way ANOVA, *p* < 0.05 for IS against NIS in all dilutions, but 3CL 1:8000. The serum of five animals was pooled for each condition.

A relevant question for the validation of an assay is to test the selectivity for the target antigen. In this way, we sought to investigate whether a nonspecific increase in IgG titer could cause false-positive detection. We performed a test to compare the detection of SARS-CoV-2 IgG antiviral antibodies by comparing sera from animals immunized with inactivated SARS-CoV-2 virus (IS) and with sera from animals immunized with other infectious agents found in Brazil, including Dengue virus (DENV) and Zika virus (ZIKV). We also tested the serum of animals immunized with Adenovirus and Rhinovirus. In these tests, we used different dilutions of serum from animals that were incubated with both antigenic targets to perform ELISA: The N protein (**Figure 2A**) and the 3CL protease (**Figure 2B**). We observed that the serum of animals immunized with ZIKV or DENV did not show cross-reactivity in this assay, and the serum from animals immunized with Adenovirus and Rhinovirus exhibited a low-intensity background signal over. Serum from non-immunized animals (NIS) was used as a control.

### The Immunoassay With Viral Antigens N Protein and 3CL Protease Allows the Detection of Anti-SARS-CoV-2 IgG Antibodies in Human Serum

**Figure 3:**
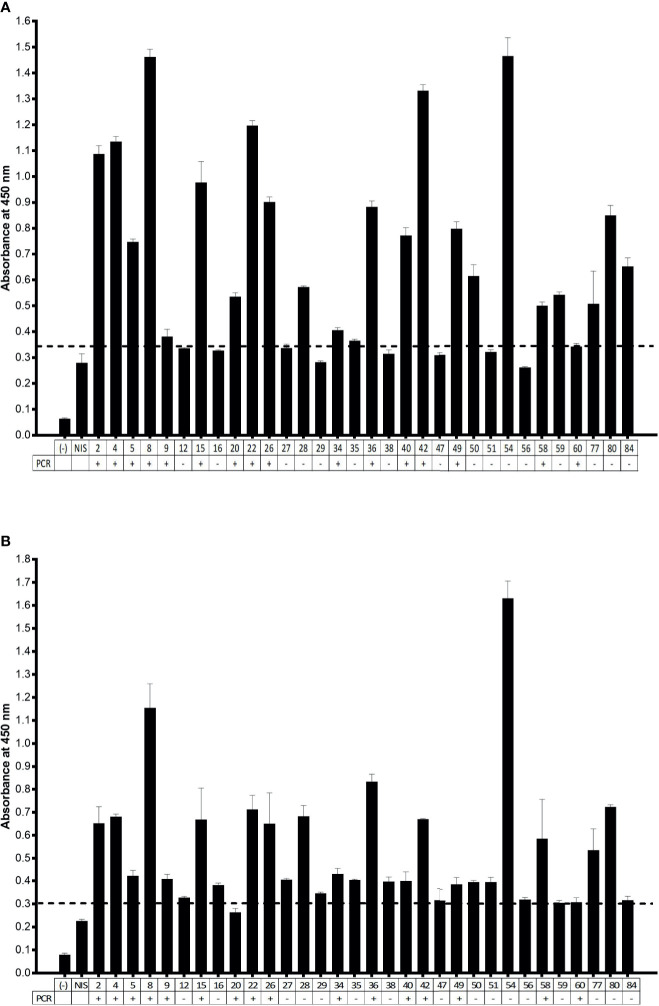
ELISA assay for IgG detection in human samples. **(A)** Serum samples diluted 1:100 were incubated to N protein-coated ELISA plates. **(B)** Serum samples diluted 1:100 were incubated to 3CL protein-coated ELISA plates. (−) negative control, absence of serum; NIS: non-immune human serum. the PCR status of nasal and oropharyngeal swab related to serum donors is indicated below the graph. Cutoff was set to NIS × 1.3. Representative experiment of two independent experiments.

After testing the ELISA with animal serum, we also tested human serum samples. These samples were collected from individuals previously diagnosed by PCR. The immunoassay consisted of revealing the presence of human anti-SARS-CoV-2 antibodies in serum samples added to plates coated with the viral antigens: N protein and 3CL protease. The dilutions of human serum were previously defined (**Supplementary Figure 1**), and we chose a dilution of 1:100, as it had a higher signal intensity under the conditions of our test. The ELISA revealed, in a semi-quantitative manner, antiviral antibodies present in the serum, compared to a non-immune serum (NIS), collected in the first semester of 2019, prior to the COVID-19 pandemic (**Supplementary Figure 6**). Thus, we could observe samples with absorbance signals above the control serum. Since we have previously collected oropharyngeal swabs from blood donors, the ELISA results could be compared with PCR results (**Figure 3**), making it possible to calculate the sensitivity and specificity of the immunoassay for both SARS-CoV-2 antigens, N protein, and 3CL protease. For this, we initially arbitrated a cutoff over the NIS absorbance, choosing a factor, which varied from 1 to 2, that allowed the calculation of sensitivity and specificity for each condition (**Supplementary Figure 2**). For serological tests, we chose a cutoff factor of 1.3, yielding a 94% sensitivity for both N and 3CL antigens, compared to PCR.

### Serological Testing on Track of Protective Antibodies

**Figure 4:**
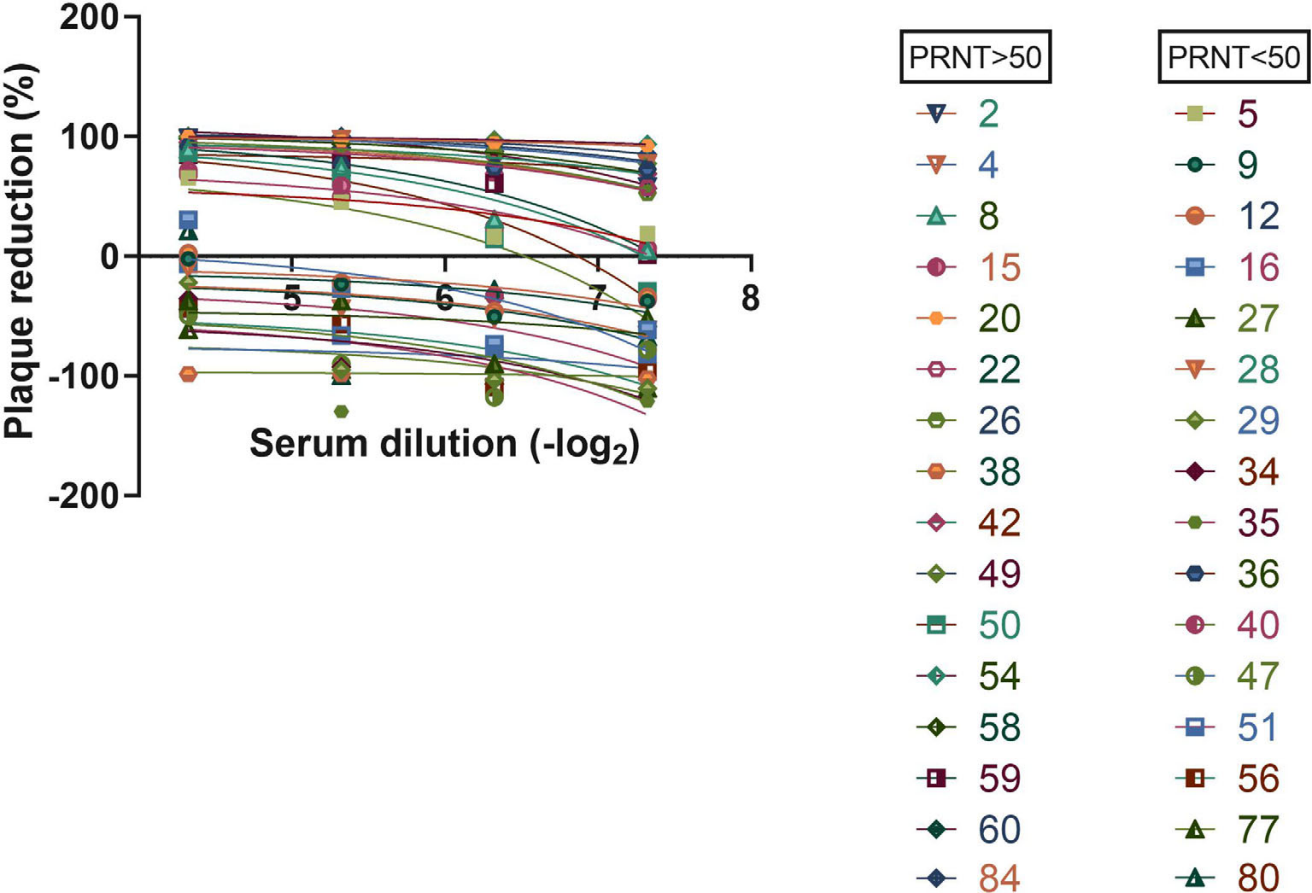
Serum neutralization assay. Virus was incubated with indicated serum dilution following cell infection. Plaque reduction was calculated.

The immunological assays allow a high-sensitivity detection of antiviral antibodies in serum samples. A relevant issue related with the use of serological assays is the determination of parameters that could suggest protective immunity mediated by neutralizing antibodies. In this sense, we investigated the potential for viral neutralization in cell culture, of human serum samples, in comparison to the results obtained with serological assays for the detection of antiviral antibodies. To test this, we performed a Plaque Reduction Neutralization Test (PRNT) with different dilutions of 32 serum samples that were characterized in our antiviral serological assays, from individuals previously tested by PCR. The PCR samples were collected 1 to 3 months before serological assay. We found that 16 samples exhibited a neutralization potential, above 50% (**Figure 4**). When analyzing ELISA’s results (**Figure 3**), we could observe high-intensity absorbance signals for some samples that stood out from the others. Thus, we arbitrated a neutralization cutoff (NCO), as being twice the detection cutoff (DCO), and in this way, we found that 79% of the samples that exhibited a high level of anti-N IgG antibodies correlated to PRNT above 50%. When analyzing results for 3CL antigen, we observed 83% of samples with PRNT above 50%, and for the combination of both N and 3CL antigens, the correlation increased to 90% of samples with PRNT above 50%.

**Figure 5:**
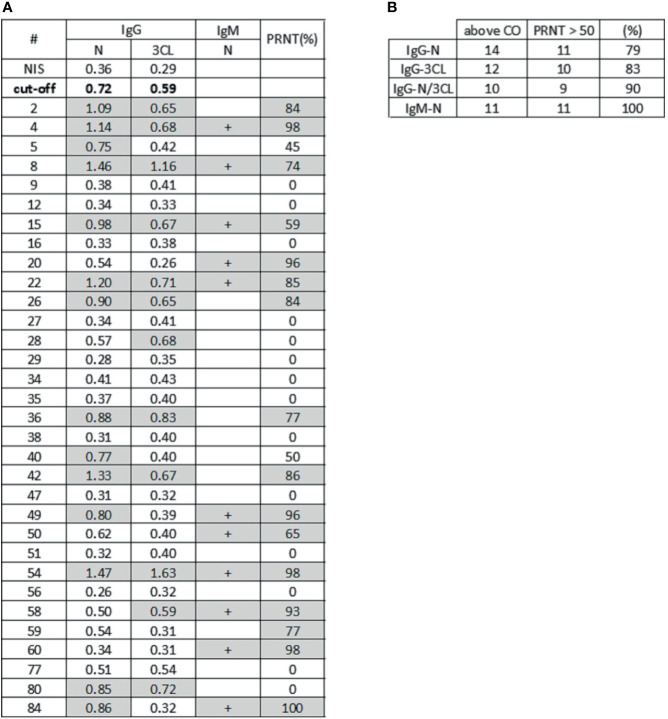
Serological profiling and virus neutralization. **(A)** The table shows absorbance signal for antiviral IgG reactive to N protein and 3CL protein by ELISA assay, IgM detection for N protein by LFA assay, and plaque reduction neutralization test (PRNT). Samples with absorbance signal above neutralization cutoff (twice NIS absorbance signal) for both antigens and samples with PRNT higher than 50% are highlighted in gray. **(B)** The table shows samples that exhibited high signal for antiviral antibodies to N protein (N), 3CL protease (3CL) and simultaneously (N and 3CL protein), in association to PRNT. In the “above CO” column, we have high IgG signal samples above the IgG positive cutoff (CO). The column PRNT>50 shows samples with neutralization activity higher than 50%. The last column shows the percentage of high neutralization samples that also have IgG signal above the cutoff, or IgM detected.

In addition to the serological immunoassays developed in our laboratory, we also used a commercial lateral flow assay (LFA) to check for the presence of antiviral IgM antibodies to SARS-CoV-2. In the LFA, we found 11 IgM-positive samples (**Figure 5**). Interestingly, we found that all these 11 IgM-positive samples also exhibited a potential for viral neutralization, with PRNT > 50%.

## Discussion

Serological testing for detection of antiviral antibodies can be used as a tool for epidemiological studies of virus circulation and evaluation of measures to contain the spread of infection. In this work, we explored immunoassays to detect antiviral antibodies that are reactive to two SARS-CoV-2 antigens, the N protein and the 3CL protease. The SARS-CoV N protein has been previously demonstrated to be a very sensitive antigen for diagnostic purposes. The antibodies generated for SARS-CoV nucleocapsid protein have been described as the most abundant compared to antibodies generated against other viral antigens (3). Immunoassays targeting SARS-CoV-2 N protein have shown high sensitivity and high specificity (6, 7, 15). Therefore, it becomes interesting to use SARS-CoV-2 N protein as an antigen to detect antiviral antibodies in serological assays. In contrast to the LFA, which indicates whether a sample is positive or negative, the ELISA also indicates the intensity of the signal, making it possible to increase the sensitivity of the test, reducing the DCO, to increase detection of low intensity signals. The ELISA may be more sensitive than LFA for detecting anti-SARS-CoV-2 antibodies (22). In some cases, we found serum samples that were positive with low signals, from individuals diagnosed positive by PCR, and that were not detected in the LFA (**Supplementary Figure 3**). In our tests, we performed the sensitivity adjustment using an arbitrary cutoff on absorbance values read for NIS. As we did not have a human antiviral antibody reactive to SARS-CoV-2 N protein that could be used to generate a standard curve for quantitative determination, we arbitrated DCO values based on the calculation of the sensitivity of the serological test compared to the previous diagnosis by PCR. These PCR tests were carried out with samples from serum donors 1 to 3 months before harvesting serum samples. Literature data also suggest that PCR tests can provide false-negative results (13, 23, 24), and in this sense, we observed that the serological test may reveal positive samples that had not been detected by PCR. We observed some PCR-negative samples that exhibited a high potential for viral neutralization (**Supplementary Figure 4**). The results of serological tests may indicate exposure to viral antigens; however, it is not completely clear whether individuals with positive serology would have a protective immunity against the SARS-CoV-2 virus. In this sense, we explored an approach using viral neutralization assays, in which cells challenged with the SARS-CoV-2 virus were incubated with human serum samples, previously analyzed in serological tests. Interestingly, we observed that simultaneous detection of a higher IgG level for two different viral antigens were associated with greater neutralization potentials. We have also observed that samples tested positive for antiviral IgM also may correlate to a higher neutralization potential. It is well known that IgM antibodies play an important role in the acute phase of infection and are usually detectable within about 2 weeks of symptoms onset (25). Despite showing a low affinity for the target, IgM antibodies have a high potential for eliminating pathogens due to their ability to activate the complement system (26). It has been previously demonstrated that IgM antibodies may activate complement system cascade to control influenza virus infections (27, 28). Interestingly, we observed that positive IgM samples, previously inactivated for the complement system, also showed a high neutralizing potential of SARS-CoV-2 in our cell culture plate reduction assays. The IgM antibodies can be up to 10,000 times more effective than IgG for mediating agglutination, which can also be an important process for viral neutralization (26). This finding may raise interesting clues that could be explored to investigate the association of IgM presence and the enhanced virus neutralization.

## Data Availability Statement

The raw data supporting the conclusions of this article will be made available by the authors, without undue reservation.

## Ethics Statement

The studies involving human participants were reviewed and approved by Research ethics Committe from Dante Pazzanese Cardiology Institute. The patients/participants provided their written informed consent to participate in this study. The animal study was reviewed and approved by Animal Ethics Committee from the Brazilian National Center for Research in Energy and Materials.

## Author Contributions

LP planned experiments, performed experiments, analyzed data and reviewed the manuscript. JT, CC, CT, RO, CB, LC, AP, RM, GL, JB, HL-W performed experiments. RM, JP-M, RV, CA, CG, and KF discussed results and reviewed the manuscript. MB supervised the study, planned experiments, analyzed data and wrote the manuscript. All authors contributed to the article and approved the submitted version.

## Conflict of Interest

The authors declare that the research was conducted in the absence of any commercial or financial relationships that could be construed as a potential conflict of interest.

## Publisher’s Note

All claims expressed in this article are solely those of the authors and do not necessarily represent those of their affiliated organizations, or those of the publisher, the editors and the reviewers. Any product that may be evaluated in this article, or claim that may be made by its manufacturer, is not guaranteed or endorsed by the publisher.
