# Supplementary material for: Serological Testing for COVID-19, Immunological Surveillance, and Exploration of Protective Antibodies
Source: Front Immunol. 2021 Aug 19;12:635701. doi: 10.3389/fimmu.2021.635701 (PMC8417107; doi:10.3389/fimmu.2021.635701)
Supplement: Supplementary file 1 [file DataSheet_1.pdf]

A

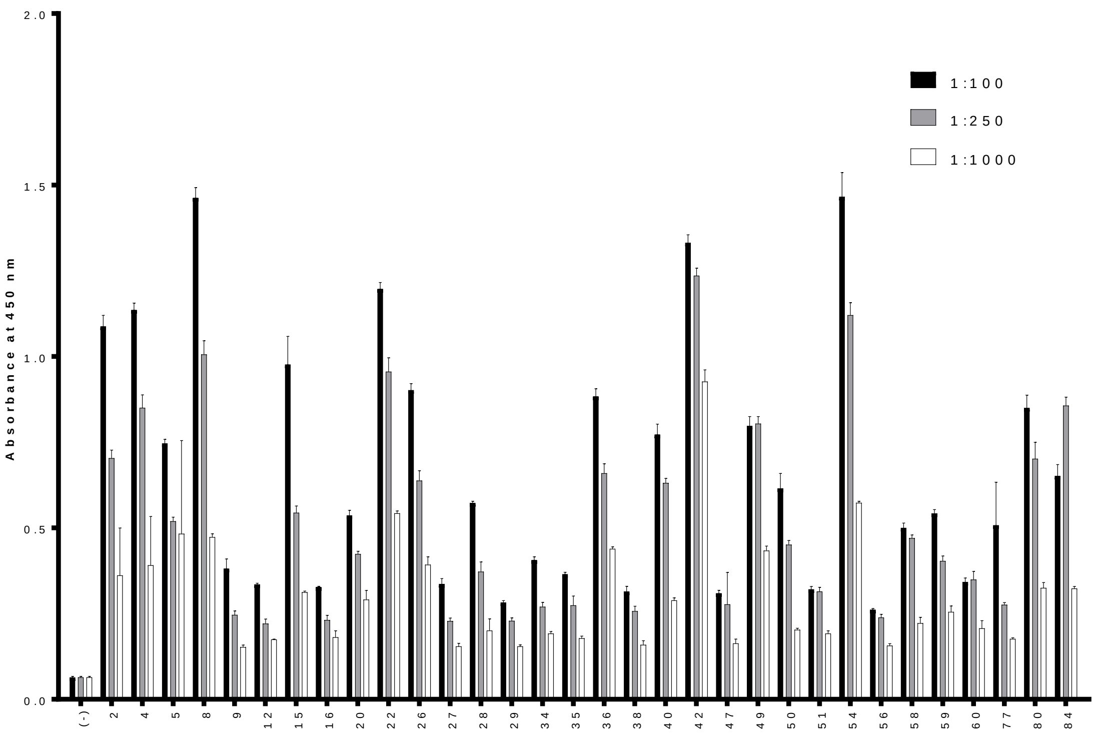

B

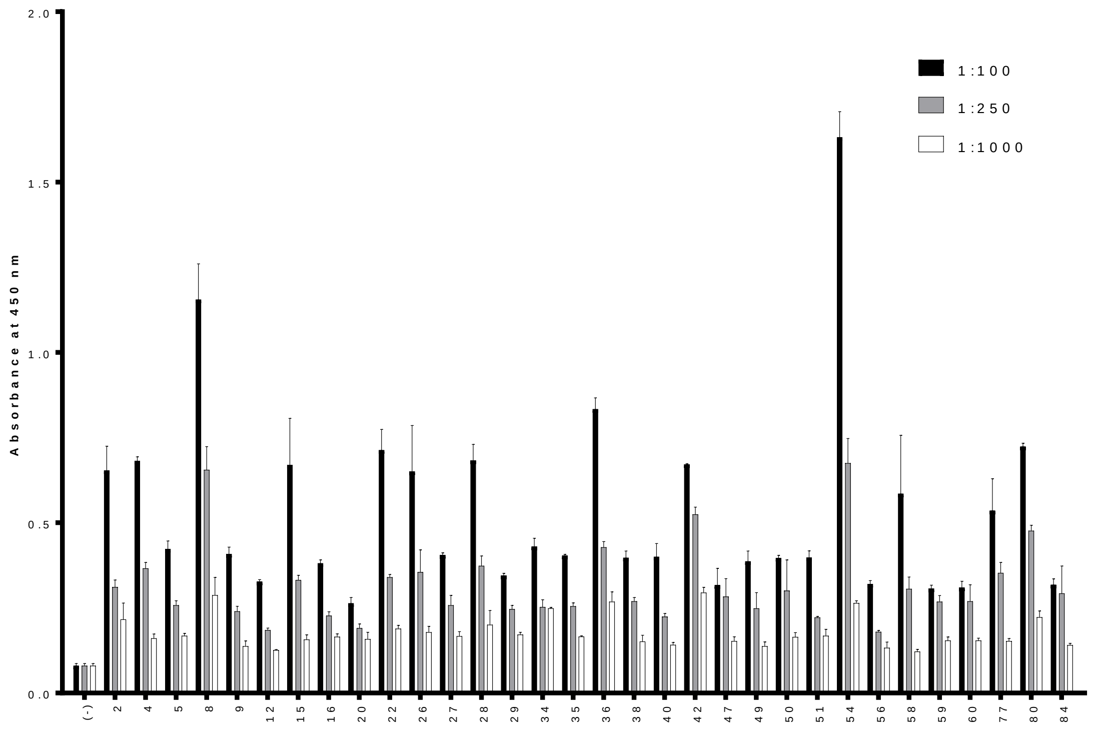

Figure S1 – ELISA using different serum dilutions. (A) N protein coated plates. (B) 3CL coated plates. (-) is the negative control reaction in the absence of serum.

cut-off	N protein		3CL protease	
factor	sens	spec	sens	spec
1	1	0.52	1	0.5
1.1	1	0.53	1	0.5
1.2	1	0.64	0.94	0.5
1.3	0.94	0.67	0.94	0.5
1.4	0.89	0.7	0.89	0.52
1.5	0.84	0.7	0.89	0.59
1.6	0.84	0.7	0.89	0.62
1.7	0.84	0.7	0.89	0.64
1.8	0.8	0.7	0.8	0.76
1.9	0.8	0.73	0.73	0.8
2	0.76	0.76	0.7	0.8

Figure S2 – Detection cut-off factor, sensitivity and specificity adjustment of ELISA. The table shows calculated sensitivity (sens) and specificity (spec) for each antigen (N protein or 3CL protease). The cut-off factor listed in the left column was applied over the value of NIS (negative immune serum) to setup detection cut-off.

#	PCR	LFA	ELISA		PRNT%
**2**	+	+	+	1,09	100
**4**	+	+	+	1,14	97
**5**	+	+	+	0,75	84
**8**	+	+	+	1,46	92
**9**	+	-	+	0,38	42
**12**	-	-	-	0,34	3
**15**	+	+	+	0,98	90
**16**	-	-	-	0,33	55
**20**	+	+	+	0,54	100
**22**	+	+	+	1,20	100
**26**	+	+	+	0,90	97
**27**	-	-	-	0,34	11
**28**	-	-	+	0,57	79
**29**	-	-	-	0,28	48
**34**	+	-	+	0,41	40
**35**	-	-	+	0,37	66
**36**	+	+	+	0,88	100
**38**	-	-	-	0,31	16
**40**	+	+	+	0,77	71
**42**	+	+	+	1,33	100
**47**	-	-	-	0,31	24
**49**	+	+	+	0,80	100
**50**	-	+	+	0,62	100
**51**	-	-	-	0,32	6
**54**	-	+	+	1,47	100
**56**	-	-	-	0,26	23
**58**	+	+	+	0,50	100
**59**	-	-	+	0,54	100
**60**	+	-	-	0,34	100
**77**	-	-	+	0,51	-2
**80**	-	-	+	0,85	42
**84**	-	+	+	0,86	100

Figure S3 – Comparison of ELISA and LFA.

#	PCR	LFA	ELISA		PRNT%
**2**	+	+	+	1,09	100
**4**	+	+	+	1,14	97
**5**	+	+	+	0,75	84
**8**	+	+	+	1,46	92
**9**	+	-	+	0,38	42
**12**	-	-	-	0,34	3
**15**	+	+	+	0,98	90
**16**	-	-	-	0,33	55
**20**	+	+	+	0,54	100
**22**	+	+	+	1,20	100
**26**	+	+	+	0,90	97
**27**	-	-	-	0,34	11
**28**	-	-	+	0,57	79
**29**	-	-	-	0,28	48
**34**	+	-	+	0,41	40
**35**	-	-	+	0,37	66
**36**	+	+	+	0,88	100
**38**	-	-	-	0,31	16
**40**	+	+	+	0,77	71
**42**	+	+	+	1,33	100
**47**	-	-	-	0,31	24
**49**	+	+	+	0,80	100
**50**	-	+	+	0,62	100
**51**	-	-	-	0,32	6
**54**	-	+	+	1,47	100
**56**	-	-	-	0,26	23
**58**	+	+	+	0,50	100
**59**	-	-	+	0,54	100
**60**	+	-	-	0,34	100
**77**	-	-	+	0,51	-2
**80**	-	-	+	0,85	42
**84**	-	+	+	0,86	100

Figure S4 – Serological assays reveal positive samples that were not detected by PCR.

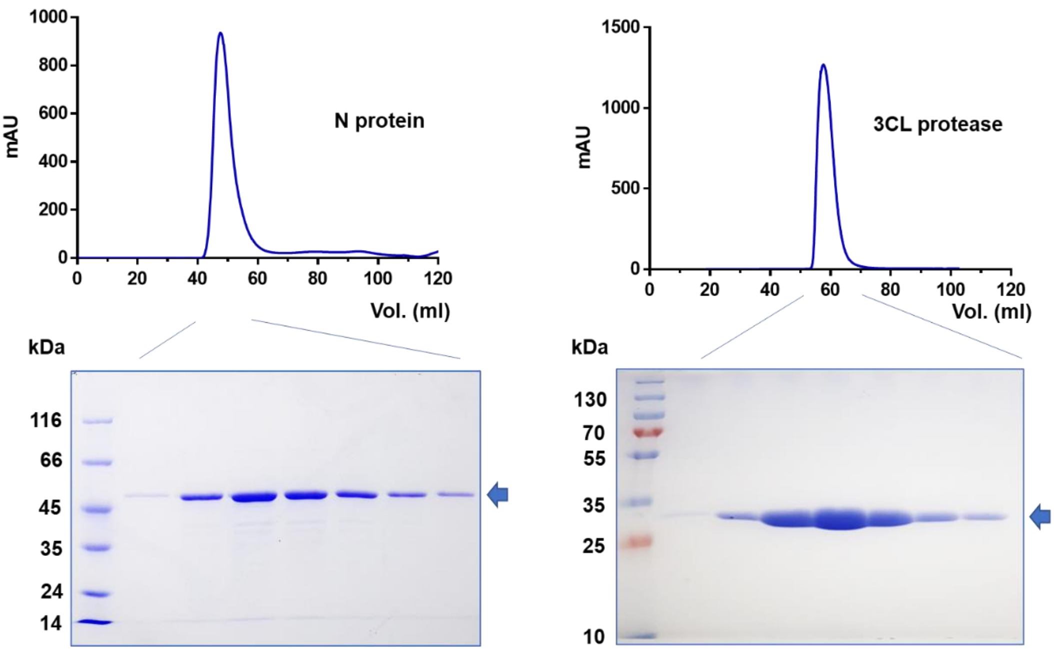

Figure S5 – The SARS-CoV-2 N and 3CL proteins as antigen. The recombinant N protein and 3CL protease produced in *E. coli* and purified by affinity chromatography were concentrated and subjected to size-exclusion chromatography, as the final purification step (upper panels). The purity of the protein fractions corresponding to the N and 3CL peaks were analyzed by SDS-PAGE (bottom panels). Arrows indicate the purified proteins used in the experiments.

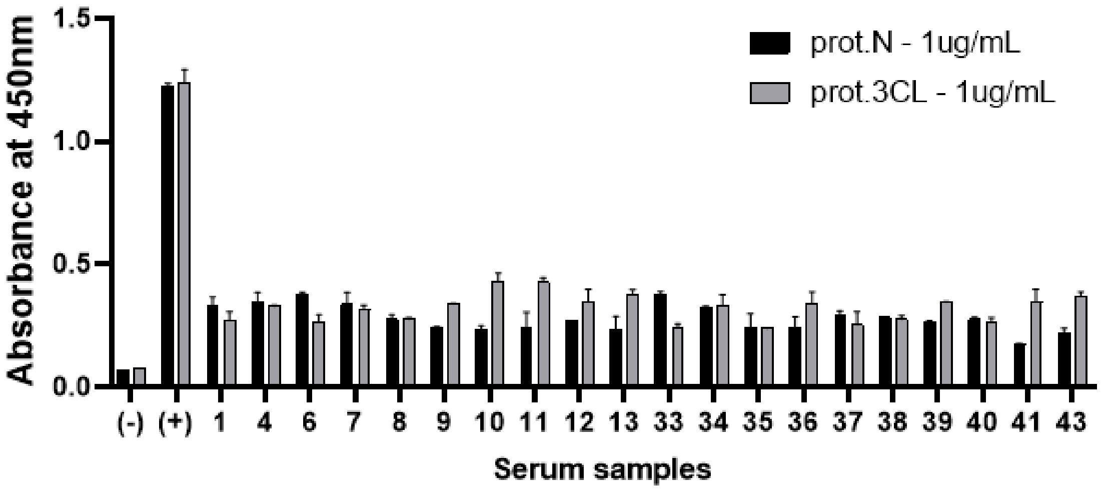

Figure S6 – ELISA of non-immune serum samples from healthy volunteers in 2019 (-) negative control; (+) positive control; Numbers from 1 to 43: human serum samples.
